# Supplementary material for: The Systems Biology Research Tool: evolvable open-source software
Source: BMC Syst Biol. 2008 Jun 29;2:55. doi: 10.1186/1752-0509-2-55 (PMC2446383; doi:10.1186/1752-0509-2-55)
Supplement: Additional file 1 — SBRT Archive. An archive of the current version of the Systems Biology Research Tool. [file 1752-0509-2-55-S1.zip › sbrt-1.4.0/doc/developers_guide/api/sbrt/kernel/math/Interval.html]

Interval


|  |  |  |
| --- | --- | --- |
| **Overview** \| **Package** \| **Class** \| **Use** \| **Tree** \| **Deprecated** \| **Index** \| **Help** | |  |
| **PREV CLASS**   **NEXT CLASS** | **FRAMES**    **NO FRAMES**     **All Classes** |  |
| SUMMARY: NESTED \| FIELD \| CONSTR \| METHOD | DETAIL: FIELD \| CONSTR \| METHOD |  |


---


## sbrt.kernel.math Class Interval

```
java.lang.Object
  sbrt.kernel.math.Interval
```

**All Implemented Interfaces:**: java.io.Serializable

---

``` public final class Interval extends java.lang.Object implements java.io.Serializable ```

This class is used to represent closed intervals
of the form [α, β], where α ≤ β.

Instances of this class are immutable.

**Author:**
:   This class was written and documented by Jeremiah Wright
    while in the Wagner lab.

**See Also:**: Serialized Form

---

**Constructor Summary**

`Interval(double lowerBound,
double upperBound)`
  
          Constructs a new interval with the provided lower and upper bounds.


| **Method Summary** | |
| --- | --- |
| `boolean` | `equals(java.lang.Object o)`             Indicates if the provided object is equal to this interval. |
| `double` | `getLower()`             Returns the lower bound (or endpoint) of this interval. |
| `double` | `getUpper()`             Returns the upper bound (or endpoint) of this interval. |
| `boolean` | `isWithin(double value)`             Indicates if the provided value lies within this interval. |
| `boolean` | `isWithin(double value, double tolerance)`             Indicates if the provided value lies within this interval, given the provided numerical tolerance. |
| `java.lang.String` | `toString()`             Returns a string representation of this interval. |

**Methods inherited from class java.lang.Object**

`clone, finalize, getClass, hashCode, notify, notifyAll, wait, wait, wait`

**Constructor Detail**

### Interval

```
public Interval(double lowerBound,
                double upperBound)
```

:   Constructs a new interval with the provided lower and upper bounds.

    **Parameters:**: `lowerBound` - the lower bound of the interval, α: `upperBound` - the upper bound of the interval, β **Throws:**: `IllegalBoundsException` - if lower bound > upper bound: `java.lang.IllegalArgumentException` - if either value is Double.NaN.


**Method Detail**

### getLower

```
public double getLower()
```

:   Returns the lower bound (or endpoint) of this interval.

    :   **Returns:**: the lower bound (or endpoint) of this interval.

---


### getUpper

```
public double getUpper()
```

:   Returns the upper bound (or endpoint) of this interval.

    :   **Returns:**: the upper bound (or endpoint) of this interval.

---


### isWithin

```
public boolean isWithin(double value)
```

:   Indicates if the provided value lies within this
    interval.
    This can be represented mathematically as:
    α ≤ `value` ≤ β.
    This is determined by applying the Java
    language numerical comparison operators
    `<` and `>` to the primitive
    `double` values. This is equivalent to calling
    `isWithin(double, double)` with a tolerance
    of `0.0d`.

    :   **Parameters:**: `value` - the numerical value to test. **Returns:**: `true` if the provided values lies within this interval; `false` otherwise.

---


### isWithin

```
public boolean isWithin(double value,
                        double tolerance)
```

:   Indicates if the provided value lies within this
    interval, given the provided numerical tolerance.
    This can be represented mathematically as:
    α - `tolerance` ≤
    `value` ≤
    β + `tolerance`.
    This is determined by applying the Java
    language numerical comparison operators
    `<` and `>` to the primitive
    `double` values.

    :   **Parameters:**: `value` - the numerical value to test.: `tolerance` - the numerical tolerance. **Returns:**: `true` if the provided values lies within this interval; `false` otherwise.

---


### equals

```
public boolean equals(java.lang.Object o)
```

:   Indicates if the provided object is equal to this
    interval. Two intervals A and B are equal if
    α_A_ = α_B_ and
    β_A_ = β_B_. This
    equality is determined by applying the Java
    language numerical comparison operator
    `==` to the primitive `double` values.

    :   **Overrides:**: `equals` in class `java.lang.Object`
    :   **Parameters:**: `o` - the object to test for equality. **Returns:**: `true` if the provided object equals this interval; `false` otherwise.

---


### toString

```
public java.lang.String toString()
```

:   Returns a string representation of this interval.

    :   **Overrides:**: `toString` in class `java.lang.Object`
    :   **Returns:**: a string representation of this interval.


---


|  |  |  |
| --- | --- | --- |
| **Overview** \| **Package** \| **Class** \| **Use** \| **Tree** \| **Deprecated** \| **Index** \| **Help** | |  |
| **PREV CLASS**   **NEXT CLASS** | **FRAMES**    **NO FRAMES**     **All Classes** |  |
| SUMMARY: NESTED \| FIELD \| CONSTR \| METHOD | DETAIL: FIELD \| CONSTR \| METHOD |  |


---
